# Functional Response (FR) and Relative Growth Rate (RGR) Do Not Show the Known Invasiveness of *Lemna minuta* (Kunth)

**DOI:** 10.1371/journal.pone.0166132

**Published:** 2016-11-18

**Authors:** Wout Van Echelpoel, Pieter Boets, Peter L. M. Goethals

**Affiliations:** 1 Laboratory of Environmental Toxicology and Aquatic Ecology, Ghent University, B-9000 Ghent, Belgium; 2 Provincial Centre of Environmental Research, B-9000 Ghent, Belgium; Stockholm University, SWEDEN

## Abstract

Growing travel and trade threatens biodiversity as it increases the rate of biological invasions globally, either by accidental or intentional introduction. Therefore, avoiding these impacts by forecasting invasions and impeding further spread is of utmost importance. In this study, three forecasting approaches were tested and combined to predict the invasive behaviour of the alien macrophyte *Lemna minuta* in comparison with the native *Lemna minor*: the functional response (FR) and relative growth rate (RGR), supplemented with a combined biomass-based nutrient removal (BBNR). Based on the idea that widespread invasive species are more successful competitors than local, native species, a higher FR and RGR were expected for the invasive compared to the native species. Five different nutrient concentrations were tested, ranging from low (4 mgN.L^-1^ and 1 mgP.L^-1^) to high (70 mgN.L^-1^ and 21 mgP.L^-1^). After four days, a significant amount of nutrients was removed by both *Lemna* spp., though significant differences among *L*. *minor* and *L*. *minuta* were only observed at lower nutrient concentrations (lower than 17 mgN.L^-1^ and 6 mgP.L^-1^) with higher nutrient removal exerted by *L*. *minor*. The derived FR did not show a clear dominance of the invasive *L*. *minuta*, contradicting field observations. Similarly, the RGR ranged from 0.4 to 0.6 d^-1^, but did not show a biomass-based dominance of *L*. *minuta* (0.5 ± 0.1 d^-1^ versus 0.63 ± 0.09 d^-1^ for *L*. *minor*). BBNR showed similar results as the FR. Contrary to our expectations, all three approaches resulted in higher values for *L*. *minor*. Consequently, based on our results FR is sensitive to differences, though contradicted the expectations, while RGR and BBNR do not provide sufficient power to differentiate between a native and an invasive alien macrophyte and should be supplemented with additional ecosystem-based experiments to determine the invasion impact.

## Introduction

Environmental degradation and biodiversity loss are considered to be important consequences of the globally increasing rate of biological invasions [1, 2]. Due to increased travel and trade, organisms are continuously transported outside their native range both intentionally and accidentally [3]. Introduction of such an alien organism in a yet non-colonized environment can, subsequently, result in its establishment and spread, thereby threatening current communities, economic activities and human health [4–6]. Consequently, the ability to forecast and impede future introductions, establishment and spread of alien species is of utmost importance to develop time- and cost-reducing measures.

Identifying potential introductions, avoiding establishment and impeding further spread of invasive alien species (IAS) by detection and subsequent large-scale eradication requires financial input and highly destructive measures [5]. As not all traits of the invader are known, new functions can be introduced without changing the community composition drastically (e.g., niche differentiation resulting in an increase in total ecosystem biomass) [7]. However, introduction of completely new traits is limited [8], underlining that knowledge and early detection is required from a conservation point of view. Understanding the process of invasion provides the possibility of counteractive management to limit invasion impact and re-establish native communities [9, 10] on a large scale. In contrast, small-scale eradication of invasive species is a more preferred action as it is less destructive and can be successfully applied when an alien species is discovered rapidly [5], though it requires a lot of time, effort and capital [11].

Forecasting invasion impact, on the other hand, is a challenge in invasion biology [9, 12, 13], as each organism interacts differently with its surrounding [4], making it hard to determine a general effect of biological invasions. With competition being theorised as one of the major mechanisms supporting successful invasion [9], several authors have been investigating the competitive interaction between native and alien species as a first signal of alien or native dominance (e.g., Vilà and Weiner [7], Njambuya *et al*. [14], Gioria *et al*. [15]). A competitive advantage depends on a difference in functional identity, which is hypothesised to be involved in determining the final impact of invasion [9, 16]. The competitive advantage is, in general, ascribed to the species with the highest functional trait value, while the intensity of the advantage is defined by the difference between the functional trait values. Therefore, approaches describing a difference in one (or more) functional trait(s) are applied to predict a species’ invasive behaviour, for instance the functional response (FR), relative growth rate (RGR), nutrient content and specific leaf area (SLA) [12, 13, 15, 17]. These differences in functional traits are also expected to be expressed at the sub-individual level (e.g., molecular, cellular, histological) allowing the application of biomarkers to identify the factors that influence invasive behaviour of closely related species [18]. Biomarkers should therefore be able to differentiate between a native and an invasive species. Despite being able to identify differences at the sub-individual level, appropriate extrapolation to population and community level remains unclear [19] and, considering a high physiological linkage, a similar response among different species is to be expected [18]. An additional drawback of this technique is the poor knowledge of appropriate biomarkers for investigating macrophyte species, when compared to biomarkers for animal research [20]. Therefore, subsequent selection of the FR and RGR is based on their reported applicability, their ease of application, their link with population and community dynamics, and their focus on either input (resource use, FR) or output (biomass production, RGR).

The functional response is a known concept in general ecology, but it is only recently introduced in invasion ecology for comparing the per-capita resource uptake rate of native and alien species in function of the resource density (e.g., Haddaway *et al*. [21], Dick, Alexander [13], Alexander *et al*. [22], Médoc *et al*. [23]). It states that an invasive alien species has a higher functional response compared to the native, because of its higher resource use efficiency [13]. In contrast to the functional response, which focuses on resource use (input-based), the relative growth rate focuses on the increase in biomass (output-based) to determine the invasion potential of an alien species and is considered as proxy for the species’ fitness [15]. Therefore, several authors have been investigating the difference in RGR between native and alien species to predict the invasion potential of an alien species (e.g., Grotkopp *et al*. [24], Njambuya, Stiers [14], Gérard *et al*. [25], Riley *et al*. [26]).

Despite a knowledge gap related to the behaviour of invasive macrophytes [27], application of the RGR to determine the invasive potential of macrophytes is rather limited to rooted macrophytes (e.g., Barrat-Segretain [28], Hussner [29], Eller *et al*. [30]), with less attention towards floating macrophytes (e.g., Netten *et al*. [31], Njambuya, Stiers [14]). Although the FR concept has proven to be successful for fish and macroinvertebrates (e.g., Alexander, Dick [22], Dodd *et al*. [32]) it has, to our knowledge, not been applied to plants. Among these floating macrophytes, duckweeds (*Lemnoideae*) are frequently occurring and well-known for their high reproduction rate and protein content [25, 33]. Consequently, their potential in treating eutrophic (waste)waters in combination with biomass production has been explored for decades (e.g., Culley *et al*. [34], Oron *et al*. [35], Hammouda *et al*. [36], Yu, Sun [33]). On the other hand, duckweed presence in natural systems is frequently characterised by dense mats that decrease light penetration and oxygen concentration, thereby negatively affecting aquatic life underneath these mats [37, 38].

In Belgium, five different *Lemna* spp. occur [39, 40], among which the native *Lemna minor* Linnaeus and the alien *Lemna minuta* Kunth. The latter is considered to be invasive in Belgium and is described as ‘widespread with a moderate impact’ (http://ias.biodiversity.be/). This offers the opportunity to test both FR and RGR on their ability to predict the invasive potential of a known invasive macrophyte.

The aim of this paper is: (1) to determine the potential applicability of the functional response for predicting a macrophyte species’ invasive potential, (2) to determine the potential applicability of the relative growth rate for predicting a macrophyte species’ invasive potential, and (3) to determine whether both approaches result in a similar conclusion or whether they provide additional information. The outcome of this research can be used to support the management of invasive alien plants and to predict their potential impact.

## Materials & Methods

### Test setup

A pure culture of *Lemna minor* was ordered from Blades Biological (UK, http://www.blades-bio.co.uk). *Lemna minuta* was collected from the Bourgoyen (51.062253, 3.673827) situated near Ghent (Belgium). Permission for *Lemna* collection was granted by the city of Ghent. About 20 fronds of each species were placed separately in plastic aquaria containing 2 L of nutrient medium based on OECD and ISO guidelines for chemical testing with *L*. *minor* and will be referred to as the modified Steinberg medium [41]. Fluorescence lamps were used to provide 16 hours of light, followed by 8 hours of darkness, with an intensity of 45–58 μmol.m^-2^.s^-1^. Temperatures of the growth medium varied between 21.6°C and 24.0°C. Every two to three days new medium was provided and aquaria were rinsed with tap water. Fronds showing the start of algae growth were removed or rinsed carefully. Selected *Lemna* spp. plants were grown in these conditions for at least two weeks to acclimate.

**Fig 1:**
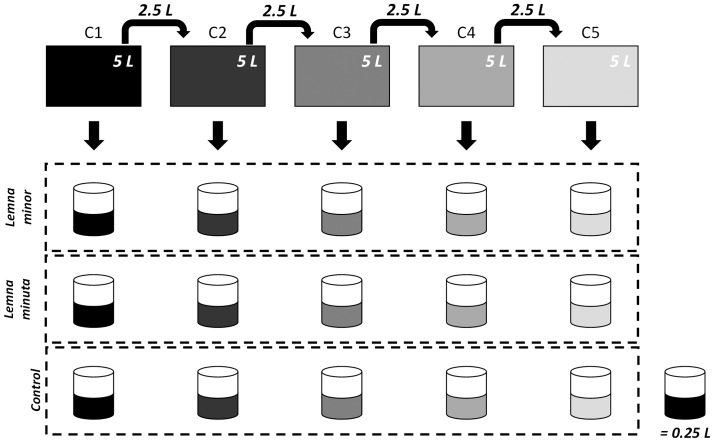
Schematic overview of the experimental set-up. Relative initial nutrient concentrations are shown on top and were sampled at the start. Darkness represents the dilution state of the growth medium (black equals original modified Steinberg medium). Each recipient was filled with 0.25 L of its respective nutrient solution and was performed in triplicate.

Tests were performed with similar light and temperature conditions as growth conditions. All recipients were covered at the sides with aluminium foil to constrain algae growth. The original modified Steinberg medium (C_0_) was diluted with deionised water to obtain the following series of concentrations: C_0_, 0.5C_0_, 0.25C_0_, 0.125C_0_, and 0.0625C_0_, from now on described as the following series: C1, C2, C3, C4, and C5. Of each concentration, 0.25 L was poured into a glass recipient and about 500 mg_fw_ of *L*. *minor* or *L*. *minuta* was added. A third series, without any plants, was created as a control. Each combination of nutrient concentration and species presence was performed in triplicate, resulting in a total of 45 recipients per test. In total, two tests were run, resulting in six replicates for each combination. A schematic overview of the experimental set-up for one single series is shown in Fig 1.

Each test lasted for four days (96 h), as preliminary experiments pointed out that nutrient concentrations were noticeably lower and algae growth was still limited. Deionised water was added daily to account for evaporation. After two days, recipients were cleansed to remove algae starting to grow on the recipient’s walls. After four days plants and growth medium were separated for further analysis. Tests were performed in October and November 2015.

### Data collection

Growth medium samples were collected at the beginning and at the end of the test and stored at 4°C in the dark prior to analysis. Within 36 hours after sampling, nutrient analysis was performed spectrophotometrically using Merck field kits for total nitrogen (test kits 1.14963.0001 and 1.14773.0001, operational range from 0.5 to 20 mgN.L^-1^) and total phosphorus analysis (test kit 1.14541.0001, operational range from 0.05 to 5 mgP.L^-1^). For each batch of samples, a blank and standard were used to determine the background signal and overall destruction efficiency, respectively. Medium samples of C1, C2, and C3 were diluted ten times with deionised water in order to comply with the operational range of the test kits. Each sample was measured thrice of which the average value represents the nutrient concentration for further calculations.

Initial dry weight content was determined by drying representative subsamples of both *L*. *minor* and *L*. *minuta* for at least 48 hours at 60°C. After two days, plant total fresh weight was determined and adapted to about 500 mg_fw_ in each sample, as to keep biomass as constant as possible (FR is considered as the per-capita resource uptake). Leftover biomass was weighed and dried (48 hours at 60°C) to determine the dry weight content and the estimated overall dry weight after two days of exposure. After four days, *Lemna* plants were harvested to determine both fresh weight and dry weight (48 hours at 60°C).

### Calculating characteristic values

Based on the obtained nutrient concentrations, nutrient mass (expressed as mgN or mgP) was derived by taking into account the volume of growth medium (0.25 L). Nutrient removal was determined as the difference in initial and final nutrient mass. For this, the initial nutrient mass was determined as the average of all six replicates per concentration. Finally, the functional response (nutrient mass removed in function of initial nutrient concentration) was determined.

Next to the absolute nutrient removal, relative nutrient removal was calculated (Eq 1), resulting in a relative nutrient removal for each individual sample.

$$R=\frac{\left(m_{0,avg}-m_{4}\right)}{m_{0,avg}}\cdot 100\%$$ (1)

With *R* the relative nutrient removal (%), *m*_*0*,*avg*_ the average nutrient mass at day 0 (mg) and *m*_*4*_ the nutrient mass at day 4 (mg).

The (estimated) dry biomass after exposure was determined after two and four days and compared with the initial (at day 0) and adapted (at day 2) dry weights, respectively. Similar to the observed nutrient removal, biomass increase was expressed both in absolute (dry weight increase) and relative (relative growth rate) terms of which the latter was calculated based on Eq 2, representing the relative growth rate (RGR) between day 2 and day 4.

$$RGR=\frac{\text{ln}DW_{4}-\text{ln}DW_{2}}{t}$$ (2)

With *RGR* the relative growth rate (d^-1^), *DW*_*4*_ the dry weight after four days (mg), *DW*_*2*_ the adapted dry weight after two days (mg) and *t* the time interval (d).

Subsequently, nutrient removal and biomass increase were combined in a single variable to determine a more species-specific nutrient removal. Nutrient removal was expressed per gram biomass, with the latter being rather dynamic, resulting in three different values: initial dry weight, final dry weight and net dry weight increase. Both initial and final dry weight were used to determine the range of nutrient removal rate, for which time was included (mgN.gDW^-1^.d^-1^ and mgP.gDW^-1^.d^-1^). The net dry weight increase was used under the assumption that duckweed allocates nutrients directly for new biomass instead of enriching already existing biomass [42]. This suggests that an increase in nutrient uptake is related to an increase in biomass production. Follow-up of this nutrient uptake per gram newly created biomass allows to determine whether new biomass has a continuous nutrient content or whether additional nutrients are stored. A species with a higher storage capacity has an advantage towards future disturbances. To determine this biomass-based nutrient removal (BBNR), Eq 3 was applied.

$$BBNR=\frac{m_{0,avg}-m_{4}}{\left(DW_{4}-DW_{2,ad}\right)+\left(DW_{2}-DW_{0}\right)}$$ (3)

With *BBNR* the biomass-based nutrient removal (mg.mgDW^-1^), *m*_*0*,*avg*_ the average initial nutrient mass (mg), *m*_*4*_ the final nutrient mass (mg), *DW*_*4*_ the biomass dry weight after four days (mg), *DW*_*2*,*ad*_ the estimated biomass dry weight at the beginning of the second period of two days (mg), *DW*_*2*_ the estimated biomass dry weight at the end of the first two days (mg) and *DW*_*0*_ the estimated initial biomass dry weight (mg).

### Statistical analysis

Obtained data of both tests were merged into one dataset and subsequently analysed using MS^®^ Excel^®^ and R [43]. Outliers were identified by Cleveland dotplots and boxplot construction [44], though were not removed from the dataset for subsequent statistical analysis. Not removing any value from the dataset was based on the fact that all analyses were performed by the authors and that randomisation was applied when possible, thereby limiting the amount of valid arguments for outlier removal. During a second run, extreme values were removed to investigate their influence on the reported results.

Secondly, normality was tested using the Shapiro-Wilk test. When no significant difference from the normal distribution was observed (*p* > 0.05), paired Student’s *t*-tests were performed, in all other cases (*p* < 0.05) the paired Wilcoxon signed-rank test was applied. All *p*-values were considered as part of a multiple comparison setup, for which a correction of the significant threshold value is required. This correction is necessary as multiple comparisons increase the odds of observing a significant difference, though it increases the possibility of a type II error (accepting null hypothesis while alternative hypothesis is correct) [45]. In short, a Bonferroni correction was applied for determining a new threshold value for each batch of five comparisons (i.e. α = 0.01).

## Results

### Nutrient removal

Nutrient analyses performed at day 0 and day 4 resulted in the average nutrient concentrations provided in Supporting Information (S1 and S2 Tables) for total nitrogen (TN) and total phosphorus (TP), respectively. Recovery of a standard solution ranged from 93 to 99% for nitrogen and from 95 to 98% for phosphorus.

As the initial nitrogen concentration of C5 (i.e. 4.22 ± 0.04 mgN.L^-1^) was already quite low, measurements of the final nitrogen concentrations happened to be below the detection limit of 0.5 mgN.L^-1^. These results were set to zero prior to determining average nitrogen concentration.

**Fig 2:**
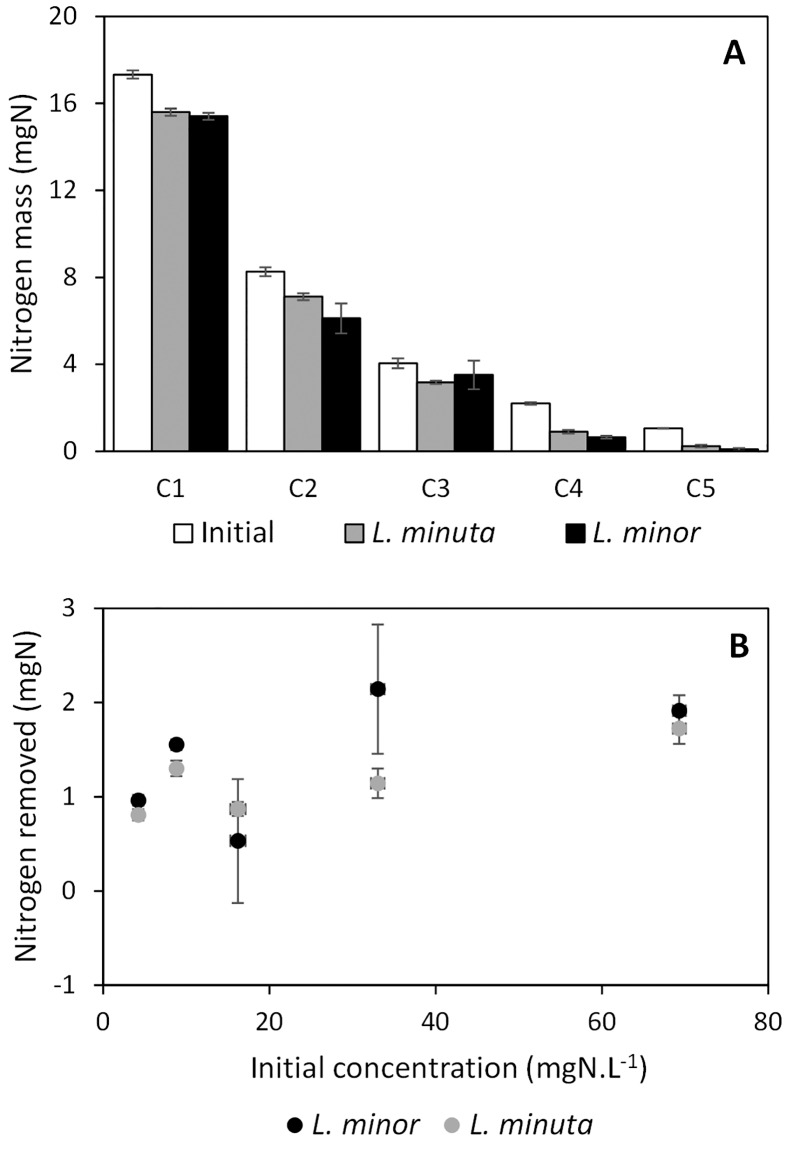
Absolute nitrogen removal by *L*. *minor* (black) and *L*. *minuta* (grey). A: nitrogen mass present at beginning (white bars) and after four days (grey and black bars). B: amount of nitrogen removed in function of the initial amount of nitrogen, representing the functional response.

**Fig 3:**
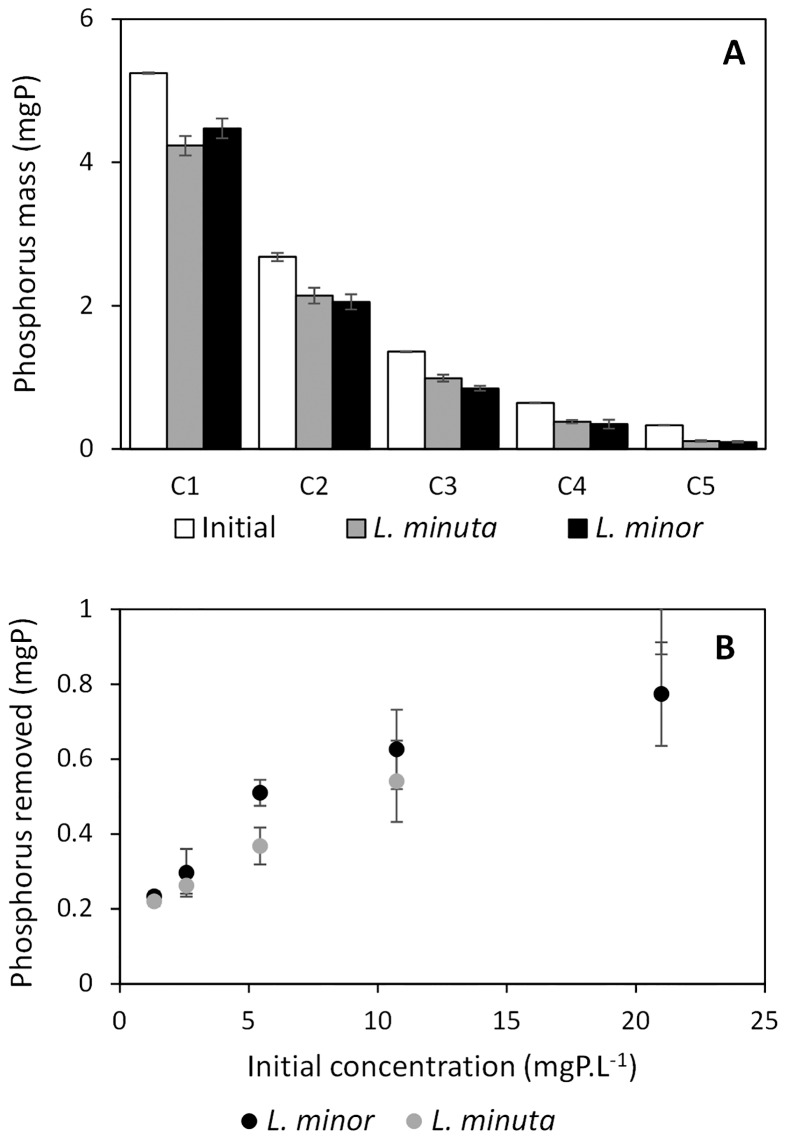
Absolute phosphorus removal by *L*. *minor* (black) and *L*. *minuta* (grey). A: phosphorus mass present at beginning (white bars) and after four days (grey and black bars). B: amount of phosphorus removed in function of the initial amount of phosphorus, representing the functional response.

Subsequently, nutrient masses (expressed in mgN and mgP) were inferred from the measured nutrient concentrations (volume of 0.25 L), resulting in a similar nutrient content for *L*. *minor* and *L*. *minuta* (see Figs 2 and 3). Both total nitrogen and total phosphorus differed significantly (*p*-values < 0.01) from the initial mass when *L*. *minor* or *L*. *minuta* was present at high (concentration C1) or low (concentration C5) nutrient concentrations (see Table 1). At intermediate concentrations, both significant and non-significant differences were observed (see Table 1). The reference series (i.e. no plants) did not show a significant difference (all *p*-values > 0.01) for nitrogen mass, though some series (C1 and C2) showed a significant difference (*p*-values < 0.01) for phosphorus mass,. Correcting for the analysis efficiency (based on the recovery of a standard solution), however, resulted in *p*-values not exceeding the threshold level of 0.01. Consequently, it can be stated that, in general, the presence of both *Lemna minor* and *Lemna minuta* after four days significantly affects the nutrient content of the provided growth medium.

**Table 1:** Obtained *p*-values after comparing initial and final nutrient masses. Significant differences (*p* < 0.01) can be found at high (C1) and low (C5) nutrient concentrations and at several intermediate nutrient concentrations.

	Nitrogen		Phosphorus	
	*L*. *minor*	*L*. *minuta*	*L*. *minor*	*L*. *minuta*
C1	< 0.001	< 0.001	0.002	< 0.001
C2	0.031	0.031	0.001	0.031
C3	0.31	0.007	< 0.001	< 0.001
C4	< 0.001	< 0.001	0.031	< 0.001
C5	< 0.001	< 0.001	< 0.001	< 0.001

**Fig 4:**
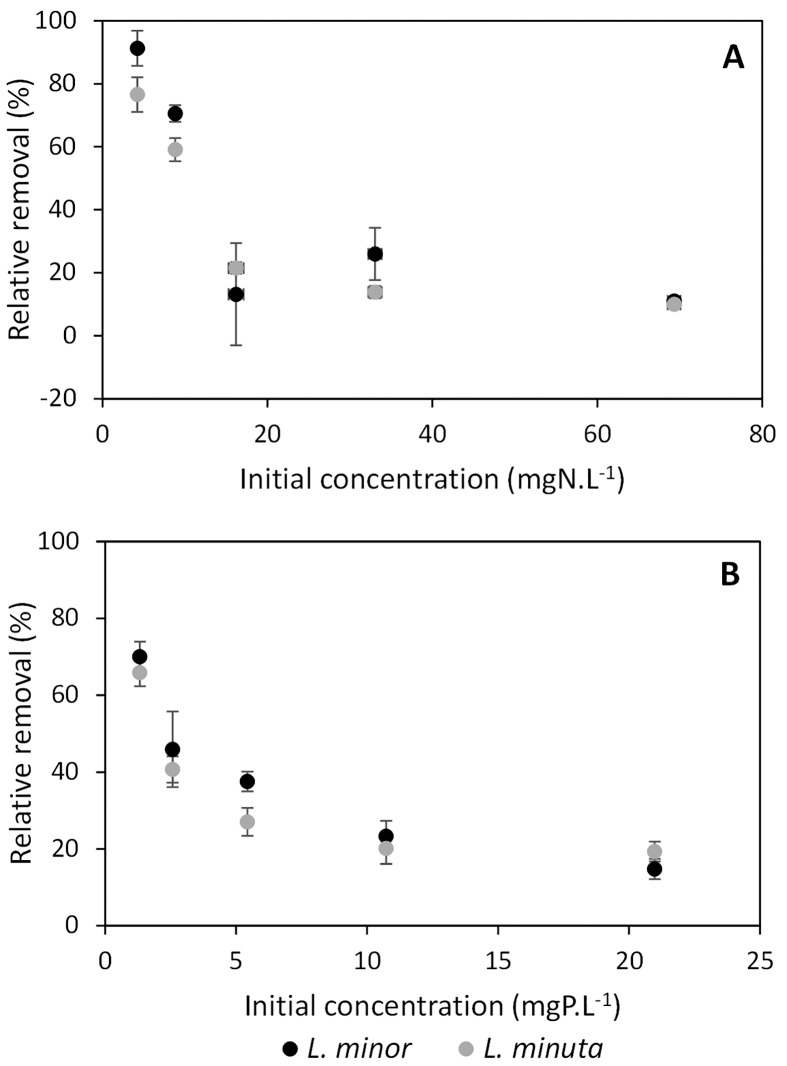
Relative removal of nutrients by *L*. *minor* (black circles) and *L*. *minuta* (grey circles). A: nitrogen removal. B: phosphorus removal. At low nutrient concentrations relatively high nutrient removal efficiencies are observed.

No significant differences in nutrient removal were found between *L*. *minor* and *L*. *minuta*, except for nitrogen at concentration C4 (*t* = -5.3557, *df* = 5, *p* = 0.003) and phosphorus at concentration C3 (*t* = -6.1281, *df* = 5, *p* = 0.002) (see Figs 2, 3 and Table 2). Relative nutrient removal, as calculated with Eq 1, showed that at low concentrations, relatively more nutrients were removed (Fig 4). Still, a slightly higher relative removal was observed for *L*. *minor* in comparison with *L*. *minuta*, with similar significant differences for nitrogen at concentrations C4 and for phosphorus at concentration C3. In short, the FR is able to identify a difference in nutrient removal, though it is limited to only one out of five concentration levels for each nutrient.

**Table 2:** Obtained *p*-values for three applied approaches showing minor similarities among the three approaches. Significant differences (*p* < 0.01) are underlined and were only observed at the nutrient level.

		C1	C2	C3	C4	C5
Concentration	Nitrogen (mgN.L^-1^)	69.3 ± 0.7	33.0 ± 0.8	16.2 ± 0.9	8.8 ± 0.2	4.22 ± 0.04
	Phosphorus (mgP.L^-1^)	20.99 ± 0.04	10.73 ± 0.06	5.43 ± 0.03	2.58 ± 0.01	1.334 ± 0.004
FR	Nitrogen	0.520	0.156	1.000	0.003	0.034
	Phosphorus	0.563	0.520	0.002	0.438	0.056
RGR		0.110	0.790	0.220	0.052	0.620
BBNR	Nitrogen	0.088	0.062	1.000	0.046	0.260
	Phosphorus	0.190	0.280	0.016	0.026	0.540

### Biomass increase

At three different moments in time (day 0, day 2 and day 4) both fresh and dry weight of *Lemna* biomass were determined, with biomass dry weight at day 0 and day 2 being estimations based on the observed dry matter content of collected subsamples. Six samples (three for each species) were removed from the dataset as not enough biomass was present to determine the dry weight content. The resulting average dry weights (estimations, except for day 4) are provided in Supporting Information (S3 and S4 Tables).

**Fig 5:**
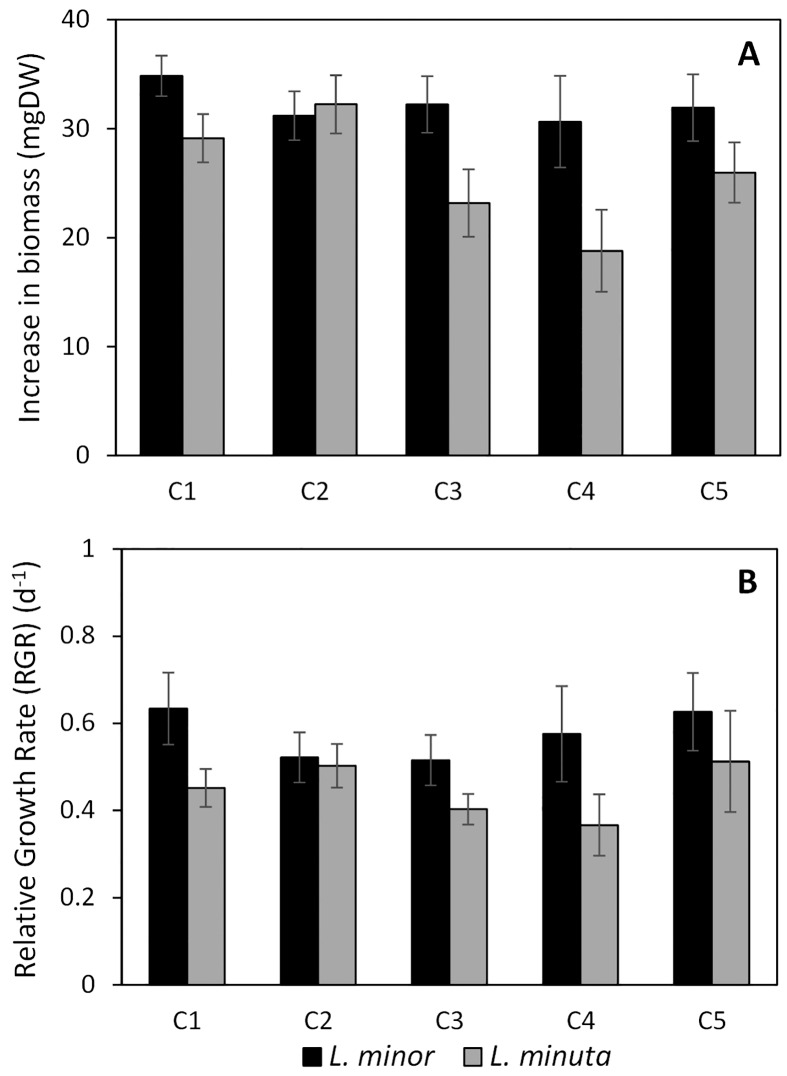
Change in biomass for *L*. *minor* (black bars) and *L*. *minuta* (grey bars). A: absolute increase in biomass dry weight (mgDW) starting from day 2 (estimation) until day 4. B: Relative Growth Rate (RGR, d^-1^) in a period of two days. Concentrations range from high (C1) to low (C5).

The increase in biomass dry weight of *L*. *minor* between day 2 and day 4 was relatively similar among different concentrations (all *p*-values > 0.01) as it ranged from 31 mgDW at concentrations C2 and C4 to 35 mgDW at concentration C1. In contrast, there was more fluctuation in the biomass increase of *L*. *minuta*, showing the highest increase (32 mgDW) at concentration C2 and the lowest increase (18 mgDW) at concentration C4 (see Fig 5), though no significant difference was observed. These fluctuations seemed to become less severe when considering the relative growth rate of *L*. *minuta*, ranging from 0.37 (± 0.07) d^-1^ at concentration C4 to 0.5 (± 0.1) d^-1^ at concentration C5 without any significant difference (all *p*-values > 0.01). In contrast, the relative growth rate of *L*. *minor* seemed to fluctuate more when compared with its related absolute biomass increase, as it ranged from 0.52 (± 0.06) d^-1^ at concentration C3 to 0.63 (± 0.08) d^-1^ at concentrations C1 and C5 (see Fig 5). Nevertheless, these growth rates were considered to be similar as no significant difference was observed (all *p*-values > 0.01).

Net biomass increase between day 2 and day 4 differed significantly between *L*. minor and *L*. *minuta* at concentration C4 (*t* = 5.3484, *df* = 4, *p* = 0.006) (Fig 5). In contrast, at concentration C2, *L*. *minor* and *L*. *minuta* were characterised by an almost identical biomass increase (*t* = -0.0772, *df* = 4, *p* = 0.942). In relative numbers however, the relative growth rate of *L*. *minor* did not differ significantly compared with *L*. *minuta* (all *p*-values > 0.01), even at concentration C4 (*t* = 2.7358, *df* = 4, *p* = 0.052). In short, the RGR did not result in a significant difference at a single concentration level and is, therefore, not able to differentiate between *L*. *minor* and *L*. *minuta*.

### Nutrient decrease versus biomass increase

Throughout the four day experiment, *L*. *minor* removed a maximum total amount of 2.1 mgN, while *L*. *minuta* removed 1.7 mgN (see also Fig 2), resulting in an approximated maximal average removal rate of 0.525 and 0.425 mgN.d^-1^, respectively. Therefore, biomass-based nitrogen uptake rates were situated in between 2.1 mmolN.gDW^-1^.d^-1^ (lowest observed dry weight of 17.6 mg) and 0.8 mmolN.gDW^-1^.d^-1^ (highest observed dry weight of 49.1 mg) for *L*. *minor* and in between 1.5 mmolN.gDW^-1^.d^-1^ (lowest observed dry weight of 20.2 mg) and 0.6 mmolN.gDW^-1^.d^-1^ (highest observed dry weight of 47.7 mg) for *L*. *minuta*. Similarly, phosphorus was removed at a maximal average removal rate of 0.19 and 0.25 mgP.d^-1^ for *L*. *minor* and *L*. *minuta*, respectively. Resulting biomass-based phosphorus removal rates were situated between 0.4 and 0.1 mmolP.gDW^-1^.d^-1^ for both *Lemna* species.

**Fig 6:**
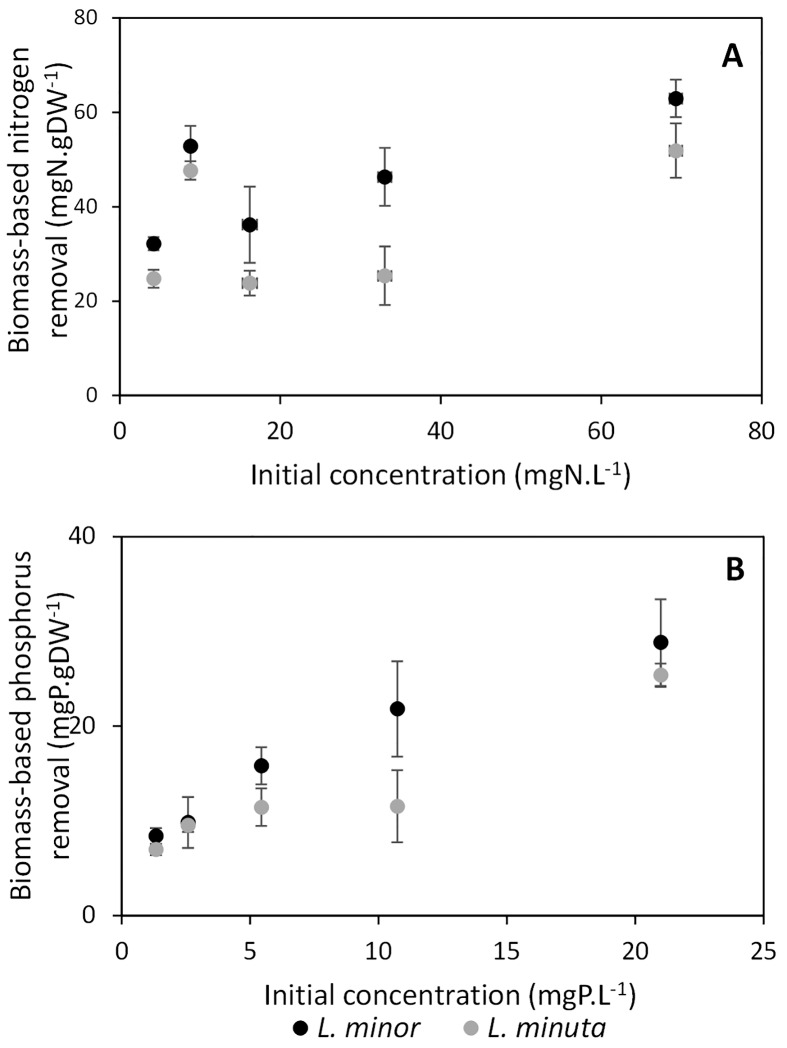
Nitrogen (A) and phosphorus (B) removal per gram newly formed biomass after four days *for L*. *minor* (black circles) and *L*. *minuta* (grey circles). Similar patterns as in Figs 2 and 3 can be observed, though differences between both *Lemna* spp. are influenced by the increase in biomass (see Fig 5).

Nutrient removal in function of biomass increase (“biomass-based nutrient removal”) varied between 20 and 65 mgN.gDW^-1^ and between 6 and 30 mgP.gDW^-1^ and combined the fluctuations in nutrient removal and biomass increase. In seemingly all cases a higher nutrient removal per gram newly formed biomass was observed for *L*. *minor*, though no significant differences were observed (all *p*-values > 0.01) (see Fig 6 and Table 2).

In short, BBNR seemed to observe similar differences in nutrient removal between *L*. *minor* and *L*. *minuta* as the FR, though it was not as powerful considering that all *p*-values were higher than the threshold value (α = 0.01) A summary of the obtained *p*-values for each of these approaches is provided in Table 2.

## Discussion

### Nutrient removal

Overall net nutrient removal by *Lemna minor* was higher than the nutrient removal exerted by *Lemna minuta* and contradicts our expectations of the latter having a higher functional response than the native *L*. *minor*. Even after removal of potential extreme values (three in total), no additional significant differences were observed. Furthermore, the difference in nutrient removal can also be noticed when considering relative nutrient removal, showing that at low nutrient concentrations both species are efficient in using the provided nutrients. This efficiency decreases with increasing concentrations, though in general, *L*. *minor* seems to be characterised by a higher resource use efficiency. These results are not in line with field observations of *L*. *minuta* dominating *L*. *minor* in Belgian water bodies. A similar contrast between field observations and experimental results was obtained when comparing two subspecies of the macrophyte *Phragmites australis*. Mozdzer *et al*. [46] clearly observed the expected pattern of higher nutrient removal by the alien subspecies, but, when applied in practice, Rodríguez *et al*. [47] observed a slightly higher nutrient removal by the native subspecies, especially towards phosphorus removal efficiency. According to Rodríguez and Brisson [47], this discrepancy can be related to the higher root biomass of the native *P*. *australis*, allowing it to take up more nutrients. This confirms both our observations and reported findings of *L*. *minor* having longer roots [14], and supports the vital role of roots in nitrogen uptake by *L*. *minor* as highlighted by Cedergreen *et al*. [48]. Additionally, these contrasting findings underline the idea that a clear difference between phylogenetically related species is hard to find and that further development and knowledge of appropriate testing methods is recommended. For instance, Colin, Porte [18] already mentioned the potential in applying biomarkers for identifying differences between native and invasive species at the sub-individual level, but also recognises the currently existing knowledge gap inhibiting its widespread application.

Our findings suggest that, despite its shown applicability at higher trophic levels (i.e. predator-prey interactions, see Dick, Alexander [13]), the functional approach does not show a higher nutrient removal by the known invader and therefore, does not allow to predict the invasive potential of *L*. *minuta*, solely based on nutrient removal. In combination with the contrasting results when comparing *Phragmites australis* [46, 47], the functional response approach does not seem to be an appropriate method in predicting the invasiveness of alien macrophytes.

### Biomass increase

In general, no significant differences were found in both absolute and relative biomass production between native and invasive *Lemna* plants. Similar to the functional response, extreme value removal (eight in total) did not result in additional significant differences with respect to the RGR. Still, *L*. *minor* seemed to perform better than *L*. *minuta*, except for condition C2, where an almost similar biomass increase was observed. This is in line with the higher observed nutrient removal by *L*. *minor* described in previous section, suggesting an overall higher efficiency in nutrient uptake by *L*. *minor*. Relative growth rates (RGR) during this period ranged from 0.5 to 0.6 d^-1^ for *L*. *minor* and from 0.4 to 0.5 d^-1^ for *L*. *minuta*. These values are higher than reported RGRs of duckweed, which are situated around 0.1 d^-1^ [14, 42] up to 0.3 d^-1^ [25, 48]. This might be related to their applied test duration of 14 to 20 days, potentially leading to overcrowding and related decrease in growth rate [49]. In contrast, Körner and Vermaat [42] only applied a duration of 3 days and observed a similarly low RGR of 0.1 d^-1^. Yet, they used domestic wastewater as a growth medium, which differs from an ideal growth medium as defined by the OECD guidelines. Nevertheless, the applied test duration of 4 days might have been too short to observe clear significant differences as nutrient concentrations might not have been depleted sufficiently to invoke a reaction at the species’ biomass level. The observed RGRs suggest that *L*. *minor* is more effective in creating new (dry) biomass. However, when focusing on fresh weight (see Supporting Information, S5 and S6 Tables), the overall biomass increase is larger for *L*. *minuta* than for *L*. *minor* (639 (± 35) mgFW versus 406 (± 19) mgFW, respectively), but a lower dry weight content reduces this difference (34 (± 1) mgDW versus 31 (± 1) mgDW, respectively). Despite the lack of clear significant differences in RGR on a dry weight basis, *L*. *minor* might still be suppressed by *L*. *minuta* producing more new, fresh biomass with a lower dry weight content. This difference in dry weight content indicates an important drawback of using RGR for dominance prediction because some field-related information is not taken into consideration. Additionally, Henry-Silva *et al*. [50] investigated the effect of nutrient conditions on three different aquatic weeds and observed that RGR on a dry weight basis does not suffice to accurately predict the infestation potential of a species. They suggest to complement the RGR data with biomass density to obtain more precise information about potential invasion problems.

In general, our findings do not fully support the idea that invasive plants have a competitive superiority over native plants due to their higher growth rates [24, 51, 52]. Moreover, our results underline the fact that comparing RGRs of monocultures only depicts the potential direct competition and neglects more important indirect competition and interactions on the long run [53]. Consequently, the relative growth rate provides information on biomass-based competition and dominance [50], though is insufficient to describe or predict the invasive potential of macrophytes as no significant differences in RGR were observed.

### Nutrient decrease versus biomass increase

Biomass-based nitrogen removal rates of both *Lemna* spp. fluctuated between 0.6 and 2.3 mmolN.gDW^-1^.d^-1^ and, thereby, included the range observed by Cedergreen and Madsen [48] for *L*. *minor* (0.6–0.9 mmolN.gDW^-1^.d^-1^). Higher maximal nitrogen removal rates were obtained by *L*. *minor* when compared to *L*. *minuta*, which might be related to the observation of *L*. *minor* plants having longer roots, potentially increasing their nutrient uptake [48]. Additionally, this difference in nutrient uptake was amplified by a higher net increase in biomass of *L*. *minuta* when compared with *L*. *minor* (Supporting Information, S3 and S4 Tables), resulting in a difference in biomass-based nutrient removal rate in favour of *L*. *minor*.

Even so, under the assumption that *Lemna* spp. reallocate nutrients for biomass increase rather than biomass enrichment [42], nitrogen contents of both *L*. *minor* and *L*. *minuta* (ranging from 20 to 63 mgN.gDW^-1^) are comparable to the values obtained by Körner and Vermaat [42], being 18.5–56.5 mgN.gDW^-1^, but are higher than reported by Cedergreen and Madsen [48], being 5.6–27.3 mgN.gDW^-1^. In contrast, phosphorus content of both *Lemna* spp. (ranging from 6 to 30 mgP.gDW^-1^) is observed to be higher than reported by Körner and Vermaat [42], being 3.6–7.2 mgP.gDW^-1^, which might be related to a difference in phosphorus content of the growth medium (1–21 mgP.L^-1^ versus 1–14 mgP.L^-1^, respectively). Duckweed is known to be a P-hyperaccumulator and to store phosphorus as a precaution to future depletion [25], which explains the increase in phosphorus removal at higher initial concentrations (see Fig 6). Nevertheless, biomass-based nutrient removal remains higher for *L*. *minor*, suggesting that *L*. *minor* requires more nutrients to produce new fronds (i.e., higher nitrogen and phosphorus content), while *L*. *minuta* biomass consists of more water. This is also supported by the observation of higher dry weight content of *L*. *minor* when compared to *L*. *minuta*.

In short, BBNR provides information about the efficiency of nutrient uptake per unit biomass, but lacks the ability to discriminate native from invasive species. Observed differences between both species were only marginally significant at the individual concentration level and were non-significant when accounting for multiple testing. Therefore, similar to FR and RGR, BBNR is not recommended to be used as the only technique to determine a macrophyte’s invasive potential, despite combining nutrient uptake and biomass increase.

### Individual traits versus ecosystem-based techniques

Combining nutrient removal (input) and biomass increase (output) does not allow to clearly differentiate between the native *L*. *minor* and invasive *L*. *minuta*. Overall, when looking at all three approaches, only two conditions were considered to be significantly different (see Table 2). Only the functional response showed a significant difference in phosphorus at concentration C3 and nitrogen at concentration C4. Firstly, this suggests that the FR is more sensitive towards differences between species, while the RGR is the least sensitive. In other words, differences are easier to be observed at the input-level than at the output-level. Secondly, the differences between *L*. *minor* and *L*. *minuta* are clearer at lower nutrient concentrations, and require further research, while the absence of significant differences at high concentrations (C1 and C2) suggests that *L*. *minor* and *L*. *minuta* have a similar nutrient removal and biomass increase. Based on these individual specific traits, the invasive character of *L*. *minuta* could not be confirmed as *L*. *minor* displayed a higher nutrient removal and a higher relative growth rate. Consequently, taking into account *L*. *minuta*’s alien origin, the increasing in-field observations and its classification as ‘widespread with moderate impact’, the applied methods are considered to be insufficient for predicting a macrophyte’s invasive potential. Nevertheless, the combined information provided by the individual traits (nutrient use and wet biomass increase) insinuates the presence of dominant behaviour of *L*. *minuta*, though this was not confirmed by the BBNR approach due to a highly fluctuating biomass increase.

Invasiveness is rarely determined by a single functional trait, but rather by a combination of factors [54]. These factors include, among others, meteorological conditions, climate, resource availability of current environment, community complexity, frequency of disturbances, phenotypic plasticity, evolutionary adaptation and predator size (see for instance, Alpert *et al*. [55], Levine, Vilà [9], Riis *et al*. [56], Gioria and Osborne [15], Baldy *et al*. [57]). Therefore, experiments applying the FR, RGR or BBNR to determine a macrophyte species’ invasive behaviour, should be supplemented with more complete and more complex ecosystem-scale research (e.g., Kovalenko *et al*. [58]). Additional attention can be given to look for appropriate biomarkers not only to study the differences between closely related species at sub-individual level, but also to increase knowledge about the existing pathways and reactions to stress. As such, both policy makers and managers can be supported by data reflecting natural conditions more accurately instead of relying on the FR, RGR or BBNR to investigate the performance of different macrophyte species with respect to nutrient removal and biomass increase.

## Conclusion

One input-based and one output-based approach were applied and supplemented with a third combined approach to test their applicability for predicting the invasive behaviour of *Lemna minuta* when compared to the native *Lemna minor*. The FR approach did not meet the expectations of a higher resource removal by the invasive species, as it was observed that *L*. *minor* removes more nutrients than *L*. *minuta*, with significant differences at low nutrient concentrations. The net dry biomass increase was higher for *L*. *minor* at low nutrient concentrations, though no significant differences were observed when comparing the RGR of both species. In contrast, the increase in fresh weight was higher for *L*. *minuta*, which supports field observations of *L*. *minuta* dominating *L*. *minor*. As such, despite not meeting the expectations of a higher FR and RGR, the low nutrient requirement and high fresh weight increase support the idea of *L*. *minuta* being more invasive than *L*. *minor*. In the observed range no dominance of the invasive alien macrophyte could be clearly inferred by applying a single approach, suggesting that other functional traits (e.g., temperature resistance, germination period, …) or environmental conditions might provide a competitive advantage [56]. Therefore, it is recommended to supplement currently existing functional traits with more in-depth and ecosystem-based research as the former, when applied individually, lacks the ability to identify and predict an invasive alien species with a moderate impact.

## Supporting Information

S1 Table Average total nitrogen (TN) concentration at day 0 and day 4 in mgN.L^-1^. (DOCX)

S2 Table Average total phosphorus (TP) concentration at day 0 and day 4 in mgP.L^-1^. (DOCX)

S3 Table Evolution of the dry weight (in mgDW) during the first two days of the experiment for *L*. *minor* and *L*. *minuta*. (DOCX)

S4 Table Evolution of the dry weight (in mgDW) during the last two days of the experiment for *L*. *minor* and *L*. *minuta*. (DOCX)

S5 Table Evolution of the fresh weight (in mgFW) during the first two days of the experiment for *L*. *minor* and *L*. *minuta*. (DOCX)

S6 Table Evolution of the fresh weight (in mgFW) during the last two days of the experiment for *L*. *minor* and *L*. *minuta*. (DOCX)
