# Supplementary material for: Functional Response (FR) and Relative Growth Rate (RGR) Do Not Show the Known Invasiveness of Lemna minuta (Kunth)
Source: PLoS One. 2016 Nov 18;11(11):e0166132. doi: 10.1371/journal.pone.0166132 (PMC5115702; doi:10.1371/journal.pone.0166132)
Supplement: S1 Table — (DOCX) [file pone.0166132.s001.docx]

**Supporting information**

**S1 Table. Average total nitrogen (TN) concentration at day 0 and day 4 in mgN.L^-1^.**

|  | Day 0 | |  | Day 4 | | | | | | | |
| --- | --- | --- | --- | --- | --- | --- | --- | --- | --- | --- | --- |
|  |  | |  | Reference | |  | *L. minor* | |  | *L. minuta* | |
| C1 | 69.3 | *(± 0.8)* |  | 70 | *(± 1)* |  | 61.6 | *(± 0.7)* |  | 62.4 | *(*± *0.7)* |
| C2 | 33.0 | *(± 0.8)* |  | 33.2 | *(± 0.6)* |  | 24 | *(± 3)* |  | 28.5 | *(*± *0.6)* |
| C3 | 16.2 | *(± 0.9)* |  | 15.2 | *(± 0.6)* |  | 14 | *(± 3)* |  | 12.7 | *(*± *0.3)* |
| C4 | 8.8 | *(± 0.2)* |  | 9.1 | *(± 0.2)* |  | 2.6 | *(± 0.2)* |  | 3.6 | *(*± *0.3)* |
| C5 | 4.22 | *(± 0.04)* |  | 4.6 | *(± 0.2)* |  | 0.4^a^ | *(± 0.2)* |  | 1.0^a^ | *(*± *0.2)* |
| ^a^ Contains samples with nitrogen concentration below detection limit. Note: Reported values are the average of six samples (two tests with three replicates each), resulting in the standard error mentioned between brackets. | | | | | | | | | | | |
