# Supplementary material for: Functional Response (FR) and Relative Growth Rate (RGR) Do Not Show the Known Invasiveness of Lemna minuta (Kunth)
Source: PLoS One. 2016 Nov 18;11(11):e0166132. doi: 10.1371/journal.pone.0166132 (PMC5115702; doi:10.1371/journal.pone.0166132)
Supplement: S2 Table — (DOCX) [file pone.0166132.s002.docx]

**Supporting information**

**S2 Table. Average total phosphorus (TP) concentration at day 0 and day 4 in mgP.L^-1^.**

|  | Day 0 | |  | Day 4 | | | | | | | |
| --- | --- | --- | --- | --- | --- | --- | --- | --- | --- | --- | --- |
|  |  | |  | Referenc*e* | |  | *L. minor* | |  | *L. minuta* | |
| C1 | 20.99 | *(*± *0.04)* |  | 20.2 | *(± 0.2)* |  | 17.9 | *(± 0.6)* |  | 16.9 | *(*± *0.5)* |
| C2 | 10.73 | *(*± *0.06)* |  | 10.2 | *(± 0.1)* |  | 8.2 | *(± 0.4)* |  | 8.6 | *(*± *0.4)* |
| C3 | 5.434 | *(*± *0.003)* |  | 5.3 | *(± 0.3)* |  | 3.4 | *(± 0.1)* |  | 4.0 | *(*± *0.2)* |
| C4 | 2.58 | *(*± *0.01)* |  | 2.48 | *(± 0.06)* |  | 1.4 | *(± 0.3)* |  | 1.53 | *(*± *0.09)* |
| C5 | 1.334 | *(*± *0.004)* |  | 1.23 | *(± 0.03)* |  | 0.40 | *(± 0.05)* |  | 0.45 | *(*± *0.05)* |
| Note: Reported values are the average of six samples (two tests with three replicates each), resulting in the standard error mentioned between brackets. | | | | | | | | | | | |
