# Supplementary material for: Functional Response (FR) and Relative Growth Rate (RGR) Do Not Show the Known Invasiveness of Lemna minuta (Kunth)
Source: PLoS One. 2016 Nov 18;11(11):e0166132. doi: 10.1371/journal.pone.0166132 (PMC5115702; doi:10.1371/journal.pone.0166132)
Supplement: S3 Table — (DOCX) [file pone.0166132.s003.docx]

**Supporting information**

**S3 Table. Evolution of the dry weight (in mgDW) during the first two days of the experiment for *L. minor* and *L. minuta*.**

|  | *L. minor* | | | | |  | *L. minuta* | | | | | |
| --- | --- | --- | --- | --- | --- | --- | --- | --- | --- | --- | --- | --- |
|  | Day 0 | |  | Day 2 | |  | | Day 0 | |  | Day 2 | |
| C1 | 22.9 | *(± 0.9)* |  | 18^a^ | *(± 3)* |  | | 20.7 | *(± 0.3)* |  | 27^a^ | *(± 2)* |
| C2 | 23.0 | *(± 0.7)* |  | 23^a^ | *(± 3)* |  | | 22 | *(± 1)* |  | 27 | *(± 3)* |
| C3 | 23.4 | *(± 0.5)* |  | 25 | *(± 3)* |  | | 20.4 | *(± 0.5)* |  | 33^a^ | *(± 1)* |
| C4 | 22.8 | *(± 0.4)* |  | 21 | *(± 4)* |  | | 20.5 | *(± 0.4)* |  | 31^a^ | *(± 5)* |
| C5 | 22.0 | *(± 0.4)* |  | 20^a^ | *(± 4)* |  | | 21.6 | *(± 0.5)* |  | 20 | *(± 10)* |
| ^a^ Dry weight content of one sample cannot be determined and is removed. | | | | | | | | | | | | |
