# Supplementary material for: Functional Response (FR) and Relative Growth Rate (RGR) Do Not Show the Known Invasiveness of Lemna minuta (Kunth)
Source: PLoS One. 2016 Nov 18;11(11):e0166132. doi: 10.1371/journal.pone.0166132 (PMC5115702; doi:10.1371/journal.pone.0166132)
Supplement: S4 Table — (DOCX) [file pone.0166132.s004.docx]

**Supporting information**

**S4 Table. Evolution of the dry weight (in mgDW) during the last two days of the experiment for *L. minor* and *L. minuta*.**

|  | *L. minor* | | | | |  | *L. minuta* | | | | | |
| --- | --- | --- | --- | --- | --- | --- | --- | --- | --- | --- | --- | --- |
|  | Day 2 | |  | Day 4 | |  | | Day 2 | |  | Day 4 | |
| C1 | 15^a^ | *(± 2)* |  | 48 | *(± 2)* |  | | 20^a^ | *(± 2)* |  | 48 | *(± 2)* |
| C2 | 18^a^ | *(± 2)* |  | 49 | *(± 2)* |  | | 19 | *(± 2)* |  | 52 | *(± 2)* |
| C3 | 19 | *(± 2)* |  | 51 | *(± 2)* |  | | 18.4^a^ | *(± 0.6)* |  | 41 | *(± 3)* |
| C4 | 16 | *(± 2)* |  | 46 | *(± 2)* |  | | 17^a^ | *(± 1)* |  | 36 | *(± 2)* |
| C5 | 14^a^ | *(± 3)* |  | 45 | *(± 2)* |  | | 17 | *(± 3)* |  | 43 | *(± 2)* |
| ^a^ Dry weight content of one sample cannot be determined and is removed. | | | | | | | | | | | | |
