# Supplementary material for: Functional Response (FR) and Relative Growth Rate (RGR) Do Not Show the Known Invasiveness of Lemna minuta (Kunth)
Source: PLoS One. 2016 Nov 18;11(11):e0166132. doi: 10.1371/journal.pone.0166132 (PMC5115702; doi:10.1371/journal.pone.0166132)
Supplement: S5 Table — (DOCX) [file pone.0166132.s005.docx]

**Supporting information**

**S5 Table. Evolution of the fresh weight (in mgFW) during the first two days of the experiment for *L. minor* and *L. minuta*.**

|  | *L. minor* | | | | |  | *L. minuta* | | | | | |
| --- | --- | --- | --- | --- | --- | --- | --- | --- | --- | --- | --- | --- |
|  | Day 0 | |  | Day 2 | |  | | Day 0 | |  | Day 2 | |
| C1 | 500.2 | *(± 0.9)* |  | 580 | *(± 30)* |  | | 500 | *(± 1)* |  | 630 | *(± 40)* |
| C2 | 499.1 | *(± 0.6)* |  | 640 | *(± 30)* |  | | 499 | *(± 0.7)* |  | 710 | *(± 20)* |
| C3 | 501 | *(± 1)* |  | 670 | *(± 20)* |  | | 499 | *(± 1)* |  | 840 | *(± 90)* |
| C4 | 500.0 | *(± 0.9)* |  | 660 | *(± 40)* |  | | 499.6 | *(± 0.9)* |  | 830 | *(± 90)* |
| C5 | 500 | *(± 1)* |  | 680 | *(± 40)* |  | | 500 | *(± 1)* |  | 750 | *(± 50)* |
|  | | | | | | | | | | | | |
